# The COVID-19 pandemic affects pregnancy complications and delivery outcomes in Japan: a large-scale nationwide population-based longitudinal study

**DOI:** 10.1038/s41598-023-48127-z

**Published:** 2023-11-29

**Authors:** Yoshiko Abe, Koji Uchiyama, Nobuko Takaoka, Keiko Yamamoto, Yasuo Haruyama, Eiji Shibata, Katsuhiko Naruse, Gen Kobashi

**Affiliations:** 1 Department of Public Health, Dokkyo Medical University, Tochigi, Japan; 2 Integrated Research Faculty for Advanced Medical Sciences, Dokkyo Medical University, Tochigi, Japan; 3 Department of Obstetrics and Gynecology, Dokkyo Medical University, Tochigi, Japan

**Keywords:** Reproductive disorders, Epidemiology, Viral infection

## Abstract

The impact of the coronavirus disease 2019 (COVID-19) pandemic on pregnancy outcomes in Japan at the national level is unclear. This study aimed to assess the impact of the pandemic on pregnancy complications and delivery outcomes in Japan using nationwide population-based longitudinal data. Secondary data from the Japan Society of Obstetrics and Gynecology from 2016 to 2020 were analyzed. Obstetric information, pregnancy complications, and delivery information of pregnant women over 22 weeks of gestation were compared before and during the pandemic. The trends of hypertensive disorder of pregnancy, fetal growth restriction, and APGAR < 7 increased, whereas those of preterm birth and low birth weight decreased during the COVID-19 pandemic. Pregnancy complications and delivery outcomes have worsened during the COVID-19 pandemic in Japan. Social changes caused by unprecedented situations may have massively influenced pregnancy in several ways. Our findings suggest that even in mild lockdowns like those in Japan, the introduction of social fear during the pandemic might negatively impact pregnancy outcomes.

## Introduction

The coronavirus disease 2019 (COVID-19) has become a global public health threat since the World Health Organization declared it a pandemic in March 2020. Many countries introduced restrictive measures, including complete lockdowns, to control the spread of the virus. Consequently, daily life and well-being were disrupted. Several studies have revealed the impact of the pandemic on our lifestyle, such as an increased snacking frequency and a craving for sweet and ultra-processed food^1^, as well as increased weight gain, body mass index (BMI), and prevalence of obesity^2^. Moreover, short-term worsening of glycemic parameters in patients with type 2 diabetes^3^, increased mortality in dementia patients without COVID-19^4^, and increased levels of psychological distress^5^ have been reported. The pandemic has also massively impacted health in Japan, with worsened HbA1c values among patients with type 2 diabetes^6^, increased psychological distress^7^, and disease exacerbation in patients with inflammatory bowel disease^8^. Moreover, due to the fear of contagion, there is a decline in the number of physician visits^9,10^, which might further deteriorate the condition.

In obstetrics, cumulative evidence has shown that the pandemic has had a profound impact on maternal, fetal, and neonatal outcomes. A recent systematic review has revealed a significant increase in stillbirths and poor maternal mental health during the pandemic. Additionally, in high-income countries, the number of preterm deliveries before a 37-week gestation and spontaneous preterm delivery has significantly decreased, and the number of surgically managed ectopic pregnancies has increased^11^. Maternal deaths have also increased in low- and middle-income countries^11,12^.

However, few studies have reported the impact of the pandemic on pregnancy outcomes in Japan. Maki et al.^13^ analyzed the rates of preterm delivery, low birth weight (LBW) infants, and small-for-gestational-age infants during the first year of the pandemic compared to those in the preceding 3 years and found no significant changes. However, the results were limited to a local area in Japan. Another study showed no significant impact on hypertensive disorder of pregnancy (HDP), threatened preterm delivery (TPD), preterm delivery, and LBW. However, these data were based on self-reported questionnaires^14^. In contrast, other single-hospital studies reported a significant reduction in the proportions of HDP, fetal growth restriction (FGR), preterm delivery, and LBW during the pandemic^15,16^. In addition, a study analyzing data from employee-based insurance in Japan found a significant reduction in pregnancy complications in 2020, such as TPD, preterm delivery, and LBW^17^. Therefore, the impact of the pandemic on pregnancy outcomes in Japan at the national level is unclear.

Given that the impact might vary based on the pandemic and restriction levels in each country or area, elucidating the impact of the pandemic on a large population in Japan is necessary for managing future public health crises. Therefore, this study aimed to assess the impact of the COVID-19 pandemic on pregnancy complications and delivery outcomes in Japan using nationwide population-based longitudinal data managed by the Japan Society of Obstetrics and Gynecology (JSOG).

## Results

### Descriptive statistics

During the past 5 years, the percentage of advanced maternal age gradually increased, while that of other age groups relatively decreased. Pregnancy with fertility treatment also increased. In 2020, vaginal delivery decreased, whereas other delivery modes such as cesarean, vacuum, and forceps showed an increasing trend. With respect to pregnancy complications, an increasing trend of HDP was observed throughout the past 5 years, and FGR increased in 2020. Regarding delivery outcomes, the percentages of each category of gestational age (weeks) at delivery and birth weight have remained almost stable in recent years. APGAR < 7, both at 1 and 5 min, increased, whereas neonatal death and stillbirth decreased in 2020. The obstetric information, pregnancy complications, and delivery outcomes from 2016 to 2020 are shown in Supplementary Table S1 online.

Table 1 shows changes in obstetric information, pregnancy complications, and delivery outcomes before and during the pandemic by the number of fetuses. The rate of advanced maternal age was higher in singleton pregnancy than in multiple pregnancy and showed a higher increase during the pandemic. Delivery in the city decreased in both singleton and multiple pregnancies and showed a greater reduction in multiple pregnancy. The rate of fertility treatment was much higher in multiple pregnancy, and it greatly increased during the pandemic. In singleton pregnancy, almost 60% underwent vaginal delivery, and it showed decreased change during the pandemic. In contrast, in multiple pregnancy, the majority were delivered via cesarean section, and it increased during the pandemic. Regarding pregnancy complications, although the rate of all complications was much greater in multiple pregnancy, both HDP and FGR showed increased change. The rates of preterm delivery and LBW were around 50% and 70% in multiple pregnancy and 11% and 15% in singleton pregnancy, respectively. Preterm delivery was decreased among singleton pregnancies, whereas it was increased among multiple pregnancy. The proportion of LBW infants was decreased in both singleton and multiple pregnancy. For APGAR < 7 and neonatal death and stillbirth, the prevalence was higher in multiple pregnancy. APGAR < 7 in 1 min was increased during the pandemic in both pregnancies. However, neonatal death and stillbirth, and maternal death were reduced in both pregnancies, and the reduction was more significant in multiple pregnancy.

**Table 1:** Differences in obstetric characteristics, pregnancy complications, and delivery outcomes between before and during the pandemic by number of fetuses.

	Before pandemic		During pandemic,		Difference
	2016–2019 (n = 955,780)		2020 (n = 201,772)		
	n	%	n	%	
Singleton pregnancy (n = 1,084,724)					
Obstetric information					
Age at deliveryª (years)					
20–34	554,131	62	114,954	61	− 0.9
≤ 19	10,058	1	1789	1	− 0.2
≥ 35	331,637	37	71,882	38	1.1
Delivery location					
City	514,573	57	108,281	57	− 0.1
Local	381,380	43	80,490	43	0.1
Pre-pregnancy BMI^a^					
Normal	540,052	71	103,924	70	0.3
Underweight	123,537	16	22,096	15	1.2
Obesity	102,585	13	22,020	15	− 1.5
Fertility treatmentª					
Yes	114,233	13	29,620	16	2.9
Mode of delivery^a^					
Vaginal	544,775	61	113,068	60	− 1.3
Emergency cesarean	126,877	14	28,381	15	0.8
Vacuum	61,051	7	13,265	7	0.2
Forceps	11,004	1	2666	1	0.2
Scheduled cesarean	145,401	16	31,217	17	0.2
Pregnancy complications					
HDPª	52,484	6	12,160	7	0.6
FGRª	32,140	4	6927	4	0.1
Delivery outcomes					
Gestational age at deliveryª					
Full term	794,152	89	167,720	89	0.2
Preterm	99,938	11	20,641	11	− 0.2
Post-term	1644	0	302	0	− 0.0
Birth weightª					
Normal	752,171	84	158,967	84	0.3
Low	135,945	15	28,068	15	− 0.3
High	7482	1	1651	1	0.0
APGAR < 7 (1 min)ª	54,703	6	12,148	6	0.3
APGAR < 7 (5 min)ª	20,390	2	4697	3	0.2
Neonatal death and stillbirthª (per 1000)	6099	7	1220	6	− 0.3
Maternal deathª (per 100,000)	71	8	13	7	− 1.0
Multiple pregnancy (n = 72,828)					
Obstetric information					
Age at delivery^b^ (years)					
20–34	37,807	63	8158	63	− 0.1
≤ 19	346	1	54	0	− 0.2
≥ 35	21,670	36	4722	37	0.3
Delivery location					
City	32,782	55	6830	53	− 2.3
Local	27,045	45	6171	48	2.3
Pre-pregnancy BMI^b^					
Normal	38,050	73	7444	72	0.3
Underweight	8335	16	1544	15	0.9
Obesity	5930	11	1295	13	− 1.3
Fertility treatment^b^					
Yes	21,832	38	5317	41	4.4
Mode of delivery^b^					
Vaginal	6004	10	1206	9	− 0.8
Emergency cesarean	23,316	39	5124	40	0.3
Vacuum	1082	2	208	2	− 0.2
Forceps	123	0	37	0	0.1
Scheduled cesarean	28,995	49	6401	49	0.6
Pregnancy complications					
HDP^b^	6350	11	1412	11	0.3
FGR^b^	5289	9	1230	10	0.6
Delivery outcomes					
Gestational age at delivery^b^					
Full term	28,346	47	6114	47	− 0.3
Preterm	31,475	53	6876	53	0.3
Post-term	2	0	3	0	0.0
Birth weight^b^					
Normal	16,313	27	3614	28	0.5
Low	43,250	73	9323	72	− 0.6
High	4	0	2	0	0.0
APGAR < 7 (1 min)^b^	7775	13	1695	13	0.0
APGAR < 7 (5 min)^b^	2937	5	635	5	− 0.0
Neonatal death and stillbirth^b^ (per 1000)	1027	17	194	15	− 2.1
Maternal death^b^ (per 100,000)	4	7	0	0	− 6.7

Missing values for number of fetuses (n = 1449) was excluded in this analysis.

*BMI* body mass index, *HDP* hypertensive disorder of pregnancy, *FGR* fetal growth restriction.

ªParticipants with missing values for age at delivery (n = 273), Pre-pregnancy BMI (n = 170,510), fertility treatment (n = 21,742), mode of delivery (n = 7019), HDP (n = 2697), FGR (n = 1318), gestational age at delivery (n = 327), birth weight (n = 440), APGAR < 7 (1 min) (n = 1835), APGAR < 7 (5 min) (n = 2853), neonatal death and stillbirth (n = 264), maternal death (n = 245) are excluded.

^b^Participants with missing values for age at delivery (n = 71), Pre-pregnancy BMI (n = 10,230), fertility treatment (n = 1821), mode of delivery (n = 332), HDP (n = 152), FGR (n = 101), gestational age at delivery (n = 12), birth weight (n = 322), APGAR < 7 (1 min) (n = 204), APGAR < 7 (5 min) (n = 247), neonatal death and stillbirth (n = 107), maternal death (n = 16) are excluded.

### Association between the pandemic and pregnancy complications and delivery outcomes

Table 2 shows the association between the pandemic and pregnancy complications and delivery outcomes by the number of fetuses. Univariate analysis showed a significant increase in HDP in singleton pregnancy. These significant results were consistent after adjusting for obstetric information (adjusted odds ratio: 1.064 [95% CI 1.040–1.090]). In multiple pregnancy, univariate analysis showed significantly increased FGR. These significant results were also consistent after adjustment of obstetric information (1.112 [1.036–1.195]). With respect to delivery outcomes, in singleton pregnancy, univariate analysis showed significant reduction of preterm delivery and LBW and an increase in APGAR < 7 (both 1 and 5 min). After adjusting for relevant factors, the associations of the pandemic with preterm delivery, LBW and APGAR < 7 (1 and 5 min) remained (0.958 [0.941–0.977] in preterm; 0.959 [0.943–0.976] in LBW, 1.030 [1.004–1.056] in APGAR < 7 [1 min]; 1.043 [1.002–1.086] in APGAR [5 min]). There were no significant association of neonatal death and stillbirth and maternal death. In multiple pregnancy, there were no significant association between the pandemic and delivery outcomes.

**Table 2:** Impact of the pandemic on pregnancy complications and delivery outcomes by number of fetuses.

	During (n = 955,780) vs. Before (n = 201,772) the pandemic					
	Univariate analysis			Multivariate analysis		
	cOR^a^	95% CI	p value	aOR^a^	95% CI	p value
Singleton pregnancy (n = 1,084,724)						
Pregnancy complications						
HDP (n = 1,082,027)	1.107	1.085–1.130	< 0.001^b^	1.064^f^	1.040–1.090	< 0.001^b,d^
FGR (n = 1,083,406)	1.026	0.999–1.053	0.059^b^	1.027^g^	0.996–1.058	0.085^b,d^
Delivery outcomes						
Gestational age at delivery (n = 1,084,397)						
Preterm	0.978	0.963–0.994	0.006^c^	0.958^h^	0.941–0.977	< 0.001^c,d^
Post-term	0.870	0.769–0.983	0.026^c^	0.835^h^	0.725–0.961	0.012^c,d^
Birth weight (n = 1,084,284)						
Low	0.977	0.963–0.991	0.001^c^	0.959^h^	0.943–0.976	< 0.001^c,d^
High	1.044	0.990–1.102	0.114^c^	1.015^h^	0.956–1.079	0.618^c,d^
APGAR < 7 (1 min) (n = 1,082,889)	1.039	1.022–1.055	< 0.001^b^	1.030^i^	1.004–1.056	0.025^b,d^
APGAR < 7 (5 min) (n = 1,081,871)	1.082	1.055–1.109	< 0.001^b^	1.043^i^	1.002–1.086	0.038^b,d^
Neonatal death and stillbirth (n = 1,084,460)	0.950	0.894–1.011	0.106^b^	0.990^i^	0.917–1.069	0.800^b,d^
Maternal death (n = 1,084,479)	0.870	0.482–1.572	0.645^b^	0.751^i^	0.318–1.775	0.514^b,d^
Multiple pregnancy (n = 72,828)						
Pregnancy complications						
HDP (n = 72,676)	1.027	0.966–1.092	0.392^b^	0.962^f^	0.897–1.031	0.272^b,e^
FGR (n = 72,727)	1.080	1.011–1.152	0.021^b^	1.112^g^	1.034–1.195	0.004^b,e^
Delivery outcomes						
Gestational age at delivery (n = 72,816)						
Preterm	1.013	0.975–1.052	0.511^c^	1.036^h^	0.992–1.081	0.110^c,e^
Post-term	6.954	1.162–41.628	0.034^c^	7.783^h^	1.295–46.782	0.025^c,e^
Birth weight(n = 72,506)						
Low	0.973	0.933–1.015	0.206^c^	1.000^h^	0.952–1.050	0.990^c,e^
High	2.257	0.413–12.327	0.347^c^	1.323^h^	0.147–11.936	0.803^c,e^
APGAR < 7 (1 min) (n = 72,624)	1.027	0.982–1.075	0.249^b^	0.996	0.931–1.066	0.915^b,e^
APGAR < 7 (5 min) (n = 72,581)	0.989	0.923–1.059	0.747^b^	0.953^i^	0.857–1.060	0.377^b,e^
Neonatal death and stillbirth (n = 72,721)	0.875	0.749–1.021	0.089^b^	0.846^i^	0.686–1.043	0.117^b,e^
Maternal death (n = 72,812)	NA	NA	NA	NA	NA	NA

Missing values for number of fetuses (n = 1449) was excluded in this analysis.

*CI* confidence interval, *HDP* hypertensive disorder of pregnancy, *FGR* fetal growth restriction.

^a^cOR and aOR mean crude and adjusted odds ratios analyzed with univariate and multivariate logistic regression models.

^b^Binary logistic regression.

^c^Multinominal logistic regression analysis.

^d^Participants with missing values for HDP (n = 191,856), FGR (n = 191,856), gestational age at delivery (n = 192,011), birth weight (n = 192,132), APGAR < 7 (1 min) (n = 199,789), AGAR < 7 (5 min) (n = 200,765), neonatal death and stillbirth (n = 198,808), and maternal death (n = 198,769) are excluded.

^e^Participants with missing values for HDP (n = 12,030), FGR (n = 12,030), gestational age at delivery (n = 12,035), birth weight (n = 12,296), APGAR < 7 (1 min) (n = 12,689), APGAR < 7 (5 min) (n = 12,728), neonatal death and stillbirth (n = 12,630), and maternal death (n = 12,604) are excluded.

^f^Adjusted for age at delivery, delivery location, pre-pregnancy BMI, fertility treatment, and FGR.

^g^Adjusted for age at delivery, delivery location, pre-pregnancy BMI, fertility treatment, and HDP.

^h^Adjusted for age at delivery, delivery location, pre-pregnancy BMI, fertility treatment, HDP, and FGR.

^i^Adjusted for age at delivery, delivery location, pre-pregancy BMI, fertility treatment, mode of delivery, HDP, FGR, gestational age at delivery, and birth weight.

Data for the facilities registered in all the years are presented in Table 3. In the univariate analysis of singleton pregnancies, HDP significantly increased during the pandemic, showing the same trend after adjusting for obstetric information (1.064 [1.039–1.091]). In contrast, the univariate analysis of multiple pregnancy showed a significant increase in FGR. These were consistent after adjustment for obstetric information (1.106 [1.027–1.192]). Regarding delivery outcomes, univariate analysis of singleton pregnancies showed a significant decrease in LBW and an increase in APGAR < 7 (both 1 and 5 min). After adjustment, preterm and LBW showed significant reduction (0.968 [0.949–0.987] in preterm, 0.970 [0.953–0.988] in LBW) and APGAR < 7 (1 and 5 min) showed significant increase (1.042 [1.015–1.070] in APGAR < 7 [1 min]; 1.057 [1.013–1.102] in APGAR [5 min]). In multiple pregnancy, no factors were associated with delivery outcomes during the pandemic.

**Table 3:** Association of the pandemic and pregnancy complications and delivery outcomes by number of fetuses among the facilities registering in all years.

	During (n = 183,027) vs. Before (n = 816,113) the pandemic					
	Univariate analysis			Multivariate analysis		
	cOR^a^	95% CI	P value	aOR^a^	95% CI	p value
Singleton pregnancy (n = 934,371)						
Pregnancy complications						
HDP (n = 932,537)	1.113	1.090–1.137	< 0.001^b^	1.064^f^	1.039–1.091	< 0.001^b,d^
FGR (n = 933,811)	1.025	0.997–1.053	0.084^b^	1.024^g^	0.993–1.056	0.136^b,d^
Delivery outcomes						
Gestational age at delivery (n = 934,066)						
Preterm	0.985	0.969–1.001	0.072^c^	0.968^h^	0.949–0.987	0.001^c,d^
Post-term	0.911	0.803–1.033	0.146^c^	0.879^h^	0.761–1.015	0.079^c,d^
Birth weight (n = 933,984)						
Low	0.985	0.970–0.999	0.038^c^	0.970^h^	0.953–0.988	0.001^c,d^
High	1.060	1.002–1.121	0.043^c^	1.034^h^	0.970–1.101	0.306^c,d^
APGAR < 7 (1 min) (n = 932,746)	1.078	1.056–1.101	< 0.001^b^	1.042^i^	1.015–1.070	0.002^b,d^
APGAR < 7 (5 min) (n = 931,733)	1.120	1.083–1.158	< 0.001^b^	1.057^i^	1.013–1.102	0.010^b,d^
Neonatal death and stillbirth (n = 934,138)	0.974	0.914–1.039	0.424^b^	1.005^i^	0.928–1.088	0.900^b,d^
Maternal death (n = 934,151)	0.971	0.533–1.768	0.922^b^	0.848^i^	0.354–2.029	0.711^b,d^
Multiple pregnancy (n = 64,769)						
Pregnancy complications						
HDP (n = 64,684)	1.017	0.955–1.083	0.597^b^	0.945^f^	0.879–1.015	0.123^b,e^
FGR (n = 64,733)	1.081	1.011–1.156	0.023^b^	1.106^g^	1.027–1.192	0.007^b,e^
Delivery outcomes						
Gestational age at delivery (n = 64,757)						
Preterm	1.010	0.971–1.050	0.622^c^	1.032^h^	0.987–1.079	0.162^c,e^
Post-term	6.422	1.073–38.442	0.042^c^	7.005^h^	1.167–42.063	0.033^c,e^
Birth weight (n = 64,477)						
Low	0.975	0.933–1.019	0.254^c^	1.004^h^	0.954–1.056	0.885^c,e^
High	2.090	0.383–11.413	0.395^c^	1.234^h^	0.137–11.111	0.851^c,e^
APGAR < 7 (1 min) (n = 64,577)	1.014	0.957–1.075	0.631^b^	1.010^i^	0.942–1.083	0.775^b,e^
APGAR < 7 (5 min) (n = 64,536)	1.008	0.920–1.103	0.869^b^	0.966^i^	0.866–1.078	0.542^b,e^
Neonatal death and stillbirth (n = 64,664)	0.867	0.739–1.017	0.079^b^	0.844^i^	0.680–1.047	0.123^b,e^
Maternal death (n = 64,753)	NA	NA	NA	NA	NA	NA

Missing values for number of fetuses (n = 1139) was excluded in this analysis.

*CI* confidence interval, *HDP* hypertensive disorder of pregnancy, *FGR* fetal growth restriction *CI* confidence interval, *HDP* hypertensive disorder of pregnancy, *FGR* fetal growth restriction.

^a^cOR and aOR mean crude and adjusted odds ratios analyzed with univariate and multivariate logistic regression models.

^b^Binary logistic regression.

^c^Multinominal logistic regression analysis.

^d^Participants with missing values for HDP (n = 168,352), FGR (n = 168,352), gestational age at delivery (n = 168,494), birth weight (n = 168,590), APGAR < 7 (1 min) (n = 175,767), APGAR < 7 (5 min) (n = 176,733), neonatal death and stillbirth (n = 174,917), and maternal death (n = 174,879) are excluded.

^e^Participants with missing values for HDP (n = 10,843), FGR (n = 10,843), gestational age at delivery (n = 10,848), birth weight (n = 11,083), APGAR < 7 (1 min) (n = 11,442), APGAR < 7 (5 min) (n = 11,479), neonatal death and stillbirth (n = 11,389), and maternal death (n = 11,363) are excluded.

^f^Adjusted for age at delivery, delivery location, pre-pregnancy BMI, fertility treatment, and FGR.

^g^Adjusted for age at delivery, delivery location, pre-pregnancy BMI, fertility treatment, and HDP.

^h^Adjusted for age at delivery, delivery location, pre-pregnancy BMI, fertility treatment, HDP, and FGR.

^i^Adjusted for age at delivery, delivery location, pre-pregnancy BMI, fertility treatment, mode of delivery, HDP, FGR, gestational age at delivery, and birth weight.

## Discussion

Our findings showed a higher trend of HDP (singleton), FGR (mltiple), and APGAR < 7 (singleton) during the COVID-19 pandemic than before the pandemic. Meanwhile, the trends of preterm and LBW (singleton) were lower during the COVID-19 pandemic. To the best of our knowledge, this is the first study to assess the impact of the COVID-19 pandemic on pregnancy complications and delivery outcomes in Japan, using large-scale nationwide longitudinal data. Previous studies in Japan have not shown consistent results due to different sample sizes and facility characteristics. Using nationwide obstetric data in Japan, we identified results regarding the impact of the pandemic that were remarkably different compared with those of previous reports. Additionally, we showed the impact of the pandemic on delivery outcomes such as FGR, APGAR < 7, neonatal death, and stillbirth, which have not been well assessed. Furthermore, a sensitivity analysis with data from facilities constantly registered for all years from 2016 to 2020 yielded similar findings, indicating that the results are robust.

A recent systematic review and meta-analysis reported that no significant effects of HDP had been identified during the pandemic^11^. However, the results are still contradictory in Japan. Ohashi et al.^17^ analyzed data from an epidemiological receipt database and showed an increasing trend of HDP but with no significant difference, similar to our findings; in contrast, other studies detected a reduction in HDP^13–15^. Few previous studies have assessed the impact of FGR in Japan. A single-center study reported a significant decrease in 2020^16^. With respect to delivery outcomes, most previous studies have shown decreased changes in preterm births and LBWs during the pandemic^11–15,17,18^, consistent with our findings. Recent systematic reviews have reported no significant changes in asphyxia, neonatal death, stillbirth, or maternal death^11,12^. A study from a single hospital in Japan also showed no significant results of asphyxia (increased Apgar scores at both 1 and 5 min)^16^. The inconsistency between findings from other countries and our study might be due to differences in preventive measures and the epidemic circumstances of COVID-19. The spread of infection in Japan was relatively gradual in 2020, and Japan had not implemented a complete lockdown as in most other developed countries. Hence, it is not appropriate to simply compare the changes in pregnancy complications and delivery outcomes during the pandemic in Japan and other studies.

For COVID-19-infected pregnancy, a report from 60% of all delivery institutions in Japan showed that severe COVID-19 in pregnant women was associated with increased preterm births^19^. Additionally, cumulative evidence indicates that COVID-19 increases the risk of pregnancy complications such as pre-eclampsia, preterm birth, stillbirth, and low birth weight^20^. However, the number of pregnant women infected during the pandemic is small. In Japan, 1043 pregnant women had COVID-19 infection between July 2020 and June, 2021, and 5.4% of them had severe disease^19^. Therefore, COVID-19 alone may not be sufficient to explain the increased adverse outcomes. Meanwhile, the Japanese government declared a state of emergency during the pandemic, which means a unenforceable lockdown, such as a requesting to refrain from non-essential outing. Howevere, even such unenforceable lockdown, maternity checks among healthy pregnant women were reduced, attended delivery by families was restricted, and return to hometowns for delivery was refrained in many facilities following government requests. In addition, not only infected pregnant women, but also close contact pregnant women were restricted antenatal checkups until the end of the home care period^21–23^, and higher rates of anxiety and depression among pregnant women were reported during the epidemic^24,25^.Therefore, social changes have been observed since early in the pandemic and have both directly and indirectly impacted pregnancies. Morover, there is a study reporting delayed health-seeking due to canceled or postponed maternity care appointments, and avoiding or postponing hospital care visits due to fear of infection contributed to 44.7% of pregnancies with complications^26^. Reduced access to care might lead to delayed diagnosis and treatment, resulting in increased pregnancy complications such as HDP and FGR. Moreover, these social changes are associated with an increased risk of depression and anxiety^27^. According to a large-scale online survey in Japan, most pregnant women had some fear or distress at the beginning of the pandemic, such as the effect of COVID-19 on the fetus and pregnancy complications^23^. It is established that these types of distress and anxiety negatively affect pregnancy complications and delivery outcomes^28–31^. Therefore, a possible explanation for the change in adverse pregnancy outcomes in our findings is that these changes may have been driven mainly by the social changes caused by the pandemic. Aditionally, given that appropriate exercise during pregnancy is associated with a significantly reduced risk of HDP than being sedentary, a more sedentary lifestyle at home than before the pandemic might have increased the risk of HDP^32^. The reduction of preterm birth might also have been driven by changes in health-care delivery and population behaviours, and the reduction of LBW was consistent with the observed trends in preterm birth^11^. Increase in APGAR < 7 might be consistently associated with increased pregnancy complications (HDP and FGR). In our data, among those with HDP or FGR, almost 40% underwent emergency cesarean delivery, indicating that HDP and FGR might increase the risk of abnormal delivery and subsequently increase APGAR < 7.

Our study had several limitations. First, we could not analyze trends after 2021 during the ongoing pandemic because JSOG data for 2021 and beyond are not yet officially available. However, we analyzed the main results by comparing 2020 with one of the years from 2016 to 2019 (Supplementary Figs. S1 and S2) in this study. To minimize those annual changes, we pooled each cross-sectional data from 2016 to 2019 as one group. Second, the possibility of time-related bias in early 2020 exists because the spread of infection in Japan was relatively gradual in 2020, but the results did not change except for the first three months of 2020 (Supplementary Table S2). This has been the predominant state of news related to COVD-19 since the beginning of 2020, when the first news about the unknown infection in China broke. In addition, at the beginning of the pandemic, details regarding treatment and aftereffects were unknown. Even if the number of infections had been modest, such social situation could have caused great socia lconfusion. Therefore, we included all data for 2020 as “During the pandemic”. Third, because almost 70% of the JSOG data are from perinatal centers in Japan, the results of pregnancy complications and delivery outcomes might be overestimated. Hence, the generalizability of the findings might be limited. Fourth, in the 2020 JSOG data, pregnant patients with COVID-19 were not distinguished. Therefore, the data might include infected pregnancy data. However, a nationwide survey of maternity services in Japan (60% of all delivery institutions in Japan) identified 1043 pregnant women diagnosed with COVID-19 between July 2020 and June 30, 2021^19^. This was approximately 0.005% of the data for 2020. Although the period did not match our data, given that relatively few infected cases were detected beginning in 2020 in Japan, the number of cases in our data was assumed to be small. Lastly, we did not directly assess lifestyle and mental changes in pregnant women influenced by social changes after COVID-19 because the JSOG data did not have information on lifestyle and mental condition.

In conclusion, pregnancy complications and delivery outcomes have worsened in Japan during the COVID-19 pandemic. Social changes caused by unprecedented situations, such as restricted access to care, changes in behavior due to recommendations to stay at home, information confusion and social fear, may have influenced pregnancy in several ways. Our findings suggest that even in unenforceable restrictions like those in Japan, the introduction of social changes during the pandemic might negatively impact pregnancy outcomes.

## Methods

### Data source

Secondary data from the nationwide longitudinal study of JSOG was analyzed. The obstetric information, pregnancy complications, and delivery information of pregnant women over 22 weeks of gestation who delivered at a registered facility were included in the database. Details of the JSOG dataset have been previously published^33^. Informed consent of the JSOG dataset from the patients was obtained at the each facilities and it was obtained from guardians if a patient was < 18 years old. In this study, data from 2016 to 2020 was used. The variables relevant to the research objectives were extracted from the dataset and were preprocessed. Between 2016 and 2020, the total number of registered deliveries was 1,159,387. Of these, 386 were excluded because their year of delivery did not match the year of the dataset. A total of 1,159,001 delivery data were included in this study. This study was conducted in accordance with the Declaration of Helsinki and was approved by the Bioethics Committee of Dokkyo Medical University (29007a) and the Clinical Research Review Subcommittee of JSOG (2017–75-3). In this study, since we used secondary data, requirement of re-obtaing informed consent was waived by approvement from the Bioethics Committee of Dokkyo Medical University.

### Variables

Obstetric information included age at delivery, delivery location, pre-pregnancy BMI, infertility treatment, number of fetuses, and mode of delivery. Age was categorized into three groups: ≤ 19 years, 20–34 years, and ≥ 35 years, with ≥ 35 years considered as advanced maternal age. We categorized delivery locations into city and local. Based on the definition of the Ministry of Land, Infrastructure, Transport and Tourism, city included metropolitan areas: Aichi, Chiba, Hyogo, Ibaraki, Kanagawa, Kyoto, Mie, Nara, Osaka, Saitama, Tokyo. Meanwhile, local included other 36 prefectures^34^. Pre-pregnancy BMI was calculated by pre-pregnancy weight divided by height (weight (kg)/[height (m)]^2^) and categorized into normal (BMI ≥ 18.5 to < 25), underweight (BMI < 18.5), and obesity (BMI ≥ 25) based on the definition of the Japan Society for the Study of Obesity^35^. The fertility treatments included artificial insemination with the husband’s semen, intracytoplasmic sperm injection, in vitro fertilization-embryo transfer, ovulation induction, in vitro fertilization, blastocyst transfer, egg donation, and insemination. The number of fetuses was categorized into singleton or multiple, and the modes of delivery were vaginal delivery, emergency cesarean section, vacuum delivery, forceps delivery, and scheduled cesarean section. Pregnancy complications included HDPand FGR and were diagnosed by obstetricians according to the JSOG diagnostic criteria^36^. HDP was diagnosed as hypertension with systolic blood pressure ≥ 140 mmHg and/or diastolic blood pressure ≧90 mmHg during pregnancy. FGR was comprehensively judged by estimated fetal body weight ≤ -1.5SD and other clinical signs. Delivery outcomes were assessed according to gestational age (weeks) at delivery (full term: 37w0d-41w6d, preterm: 22w0d-36w6d, post-term: ≥ 42w0d), birth weight (normal: ≥ 2500 g to < 4000 g, LBW: < 2500 g, high birth weight: ≥ 4000 g), presence of APGAR < 7 at 1 min, presence of APGAR < 7 at 5 min, neonatal death and stillbirth, and maternal death.

### Statistical analysis

The data from 2016 to 2019 were labeled as before the pandemic, while the data from 2020 was used during the pandemic. Obstetric information, pregnancy complications, and delivery outcomes were described as frequencies and percentages and compared before and during the pandemic using the chi-squared test or Fisher’s exact test. Logistic regression analysis was conducted to estimate the unadjusted and adjusted odds ratios and 95% confidence intervals for pregnancy complications and delivery outcomes before and during the pandemic. The models were adjusted for different subsets of covariates depending on the outcomes. Variables used for adjustment were considered based on the different time periods which the variables containe; pre-pregnancy (pre-pregnancy BMI, fertility treatment), during pregnancy (HDP, FGR), delivery (mode of delivery, gestational age at delivery, birth weight), after delivery (APGAR < 7, neonatal death, maternal death). It means that HDP and FGR were adjusted by sociodemographic factors (age, location) and pre-pregnancy factors. Similally, gestational age at delivery and birth weight were adjusted by sociodemographic factors, pre-pregnancy factors and during pregnancy factors (HDP, FGR). APGAR < 7, neonatal death and maternal death were adjusted by sociodemographic factors, pre-pregnancy factors, during pregnancy factors and delivery factors. The analysis was stratified by the number of fetuses (singleton or multiple) to determine their influence on the outcomes. Sensitivity analysis was conducted using the data of facilities registered in all years from 2016 to 2020 to minimize bias in the characteristics of the registered facilities. Statistical significance was set at p < 0.05. All statistical analyses were performed using the IBM SPSS Statistics 27 for Windows (IBM Japan, Tokyo, Japan).

### Supplementary Information

Supplementary Figures.

Supplementary Tables.

## Data Availability

The data that support the findings of this study are available from Japan Society of Obstetrics and Gynecology but restrictions apply to the availability of these data, which were used under license for the current study, and so are not publicly available. Data are however available from the authors upon reasonable request and with permission of Japan Society of Obstetrics and Gynecology.
