# Supplementary material for: The COVID-19 pandemic affects pregnancy complications and delivery outcomes in Japan: a large-scale nationwide population-based longitudinal study
Source: Sci Rep. 2023 Nov 29;13:21059. doi: 10.1038/s41598-023-48127-z (PMC10686978; doi:10.1038/s41598-023-48127-z)
Supplement: Supplementary file 1 — Supplementary Figures. [file 41598_2023_48127_MOESM1_ESM.pptx]

## Slide 1
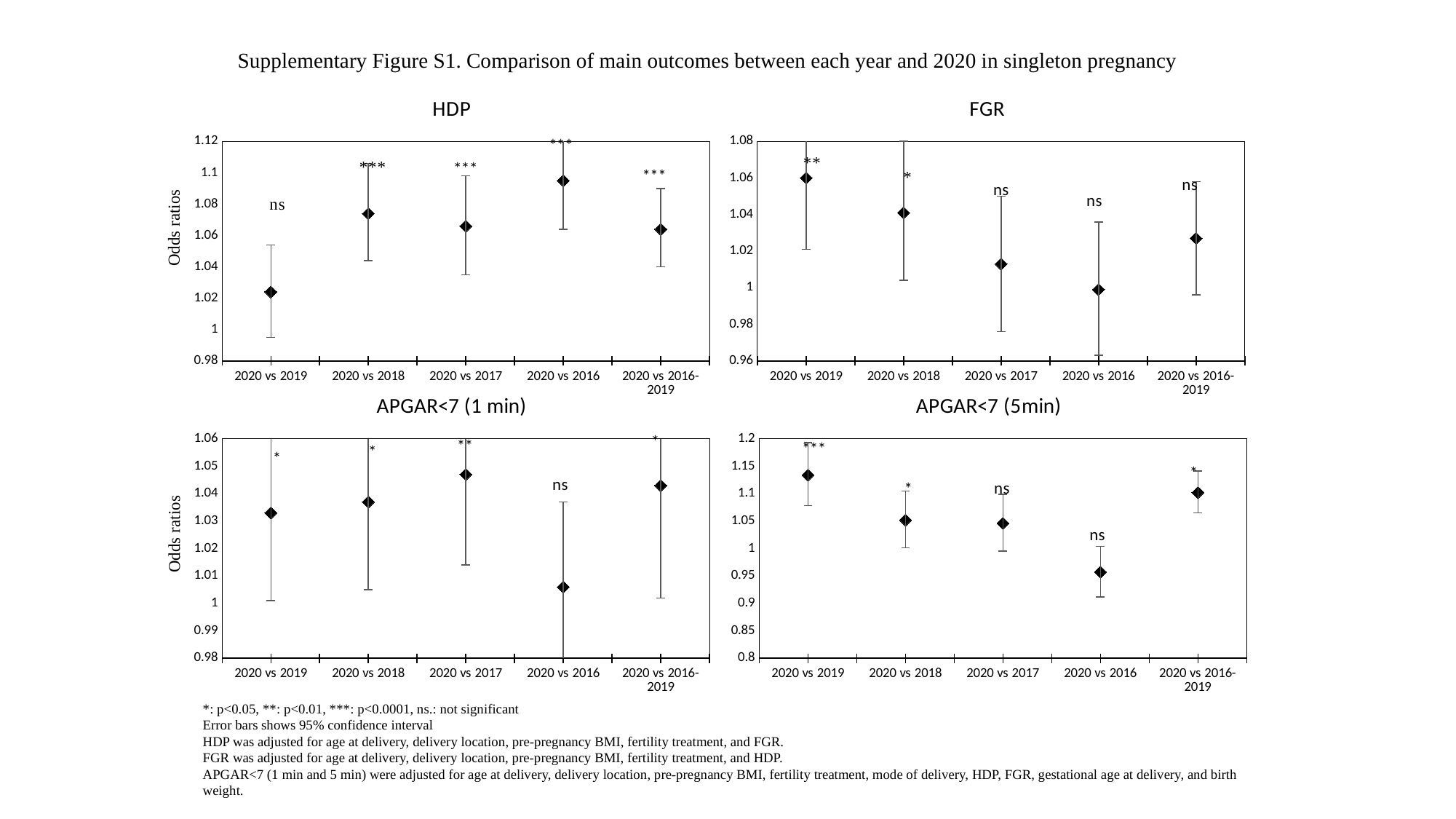

Supplementary Figure S1. Comparison of main outcomes between each year and 2020 in singleton pregnancy
### Chart: HDP
| Category | aOR |
|---|---|
| 2020 vs 2019 | 1.024 |
| 2020 vs 2018 | 1.074 |
| 2020 vs 2017 | 1.066 |
| 2020 vs 2016 | 1.095 |
| 2020 vs 2016-2019 | 1.064 |
### Chart: FGR
| Category | aOR |
|---|---|
| 2020 vs 2019 | 1.06 |
| 2020 vs 2018 | 1.041 |
| 2020 vs 2017 | 1.013 |
| 2020 vs 2016 | 0.999 |
| 2020 vs 2016-2019 | 1.027 |Odds ratios
### Chart: APGAR<7 (1 min)
| Category | aOR |
|---|---|
| 2020 vs 2019 | 1.033 |
| 2020 vs 2018 | 1.037 |
| 2020 vs 2017 | 1.047 |
| 2020 vs 2016 | 1.006 |
| 2020 vs 2016-2019 | 1.043 |
### Chart: APGAR<7 (5min)
| Category | aOR |
|---|---|
| 2020 vs 2019 | 1.134 |
| 2020 vs 2018 | 1.052 |
| 2020 vs 2017 | 1.046 |
| 2020 vs 2016 | 0.957 |
| 2020 vs 2016-2019 | 1.102 |Odds ratios
*: p<0.05, **: p<0.01, ***: p<0.0001, ns.: not significant
Error bars shows 95% confidence interval
HDP was adjusted for age at delivery, delivery location, pre-pregnancy BMI, fertility treatment, and FGR.
FGR was adjusted for age at delivery, delivery location, pre-pregnancy BMI, fertility treatment, and HDP.
APGAR<7 (1 min and 5 min) were adjusted for age at delivery, delivery location, pre-pregnancy BMI, fertility treatment, mode of delivery, HDP, FGR, gestational age at delivery, and birth weight.

## Slide 2
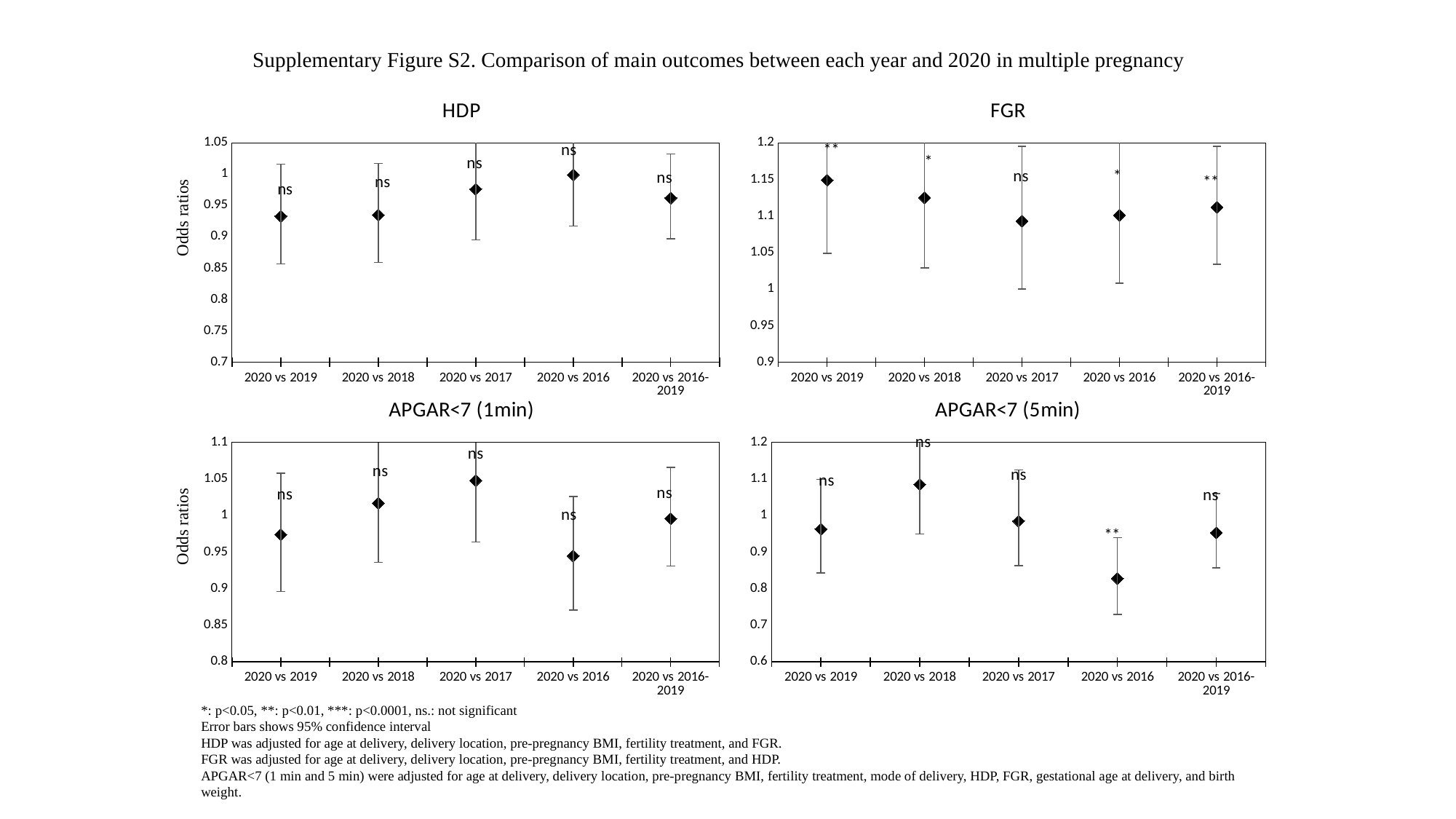

Supplementary Figure S2. Comparison of main outcomes between each year and 2020 in multiple pregnancy
### Chart: HDP
| Category | aOR |
|---|---|
| 2020 vs 2019 | 0.933 |
| 2020 vs 2018 | 0.935 |
| 2020 vs 2017 | 0.976 |
| 2020 vs 2016 | 0.999 |
| 2020 vs 2016-2019 | 0.962 |
### Chart: FGR
| Category | aOR |
|---|---|
| 2020 vs 2019 | 1.149 |
| 2020 vs 2018 | 1.125 |
| 2020 vs 2017 | 1.093 |
| 2020 vs 2016 | 1.101 |
| 2020 vs 2016-2019 | 1.112 |ns
Odds ratios
### Chart: APGAR<7 (1min)
| Category | aOR |
|---|---|
| 2020 vs 2019 | 0.974 |
| 2020 vs 2018 | 1.017 |
| 2020 vs 2017 | 1.048 |
| 2020 vs 2016 | 0.945 |
| 2020 vs 2016-2019 | 0.996 |
### Chart: APGAR<7 (5min)
| Category | aOR |
|---|---|
| 2020 vs 2019 | 0.963 |
| 2020 vs 2018 | 1.085 |
| 2020 vs 2017 | 0.985 |
| 2020 vs 2016 | 0.828 |
| 2020 vs 2016-2019 | 0.953 |Odds ratios
*: p<0.05, **: p<0.01, ***: p<0.0001, ns.: not significant
Error bars shows 95% confidence interval
HDP was adjusted for age at delivery, delivery location, pre-pregnancy BMI, fertility treatment, and FGR.
FGR was adjusted for age at delivery, delivery location, pre-pregnancy BMI, fertility treatment, and HDP.
APGAR<7 (1 min and 5 min) were adjusted for age at delivery, delivery location, pre-pregnancy BMI, fertility treatment, mode of delivery, HDP, FGR, gestational age at delivery, and birth weight.
