# Supplementary material for: The COVID-19 pandemic affects pregnancy complications and delivery outcomes in Japan: a large-scale nationwide population-based longitudinal study
Source: Sci Rep. 2023 Nov 29;13:21059. doi: 10.1038/s41598-023-48127-z (PMC10686978; doi:10.1038/s41598-023-48127-z)
Supplement: Supplementary file 2 — Supplementary Tables. [file 41598_2023_48127_MOESM2_ESM.docx]

Supplementary Table S1. Participant characteristics by year

|  |  | 2016 (n=244500) | | 2017 (n=236475) | | 2018 (n=240987) | | 2019 (n=233818) | | 2020 (n=203221) | |
| --- | --- | --- | --- | --- | --- | --- | --- | --- | --- | --- | --- |
|  |  | n | % | n | % | n | % | n | % | n | % |
| Obstetric information | | | | | | | | | | | |
| Age at deliveryª (years) | 20–34 | 151792 | 62 | 146627 | 62 | 149163 | 62 | 144356 | 62 | 123468 | 61 |
|  | ≤19 | 2919 | 1 | 2706 | 1 | 2500 | 1 | 2279 | 1 | 1844 | 1 |
|  | ≥35 | 89773 | 37 | 87132 | 37 | 89224 | 37 | 87178 | 37 | 76840 | 38 |
| Delivery location | City | 140223 | 57 | 134301 | 57 | 139375 | 58 | 133456 | 57 | 116457 | 57 |
|  | Local | 104277 | 43 | 102174 | 43 | 101612 | 42 | 100362 | 43 | 86764 | 43 |
| Pre-pregnancy BMI^a^ | Normal | 147652 | 71 | 144166 | 71 | 145986 | 71 | 140298 | 71 | 112217 | 70 |
|  | Underweight | 35734 | 17 | 33189 | 16 | 32381 | 16 | 30568 | 15 | 23832 | 15 |
|  | Obesity | 25781 | 12 | 26542 | 13 | 28061 | 14 | 28131 | 14 | 23426 | 15 |
| Fertility treatmentª | Yes | 34601 | 14 | 25943 | 11 | 37304 | 15 | 38217 | 16 | 35137 | 17 |
| Number of fetusesª | Singleton | 229337 | 94 | 221420 | 94 | 225908 | 94 | 219288 | 94 | 188771 | 94 |
|  | Multiple | 15163 | 6 | 15055 | 6 | 15079 | 6 | 14530 | 6 | 13001 | 6 |
| Mode of deliveryª | Vaginal | 142172 | 58 | 136225 | 58 | 138645 | 58 | 133737 | 57 | 115109 | 57 |
|  | Emergency cesarean | 37250 | 15 | 36683 | 16 | 38189 | 16 | 38071 | 16 | 33700 | 17 |
|  | Vacuum | 15599 | 6 | 15532 | 7 | 15682 | 7 | 15320 | 7 | 13505 | 7 |
|  | Forceps | 2780 | 1 | 2794 | 1 | 2848 | 1 | 2705 | 1 | 2720 | 1 |
|  | Scheduled cesarean | 44517 | 18 | 43265 | 18 | 43939 | 18 | 42675 | 18 | 37834 | 19 |
| Pregnancy complications | |  |  |  |  |  |  |  |  |  |  |
| HDPª | | 14372 | 6 | 14345 | 6 | 14932 | 6 | 15185 | 7 | 13582 | 7 |
| FGRª | | 9766 | 4 | 9386 | 4 | 9264 | 4 | 9013 | 4 | 8183 | 4 |
| Delivery outcomes |  |  |  |  |  |  |  |  |  |  |  |
| Gestational age at deliveryª | Full term | 210435 | 86 | 203002 | 86 | 207608 | 86 | 201453 | 86 | 175159 | 86 |
|  | Preterm | 33541 | 14 | 33013 | 14 | 32895 | 14 | 31964 | 14 | 27636 | 14 |
|  | Post-term | 454 | 0 | 416 | 0 | 425 | 0 | 351 | 0 | 307 | 0 |
| Birth weightª | Normal | 196526 | 80 | 189544 | 80 | 194186 | 81 | 188228 | 81 | 163832 | 81 |
|  | Low | 45885 | 19 | 44906 | 19 | 44814 | 19 | 43590 | 19 | 37558 | 19 |
|  | High | 1905 | 1 | 1894 | 1 | 1871 | 1 | 1816 | 1 | 1663 | 1 |
| APGAR <7 (1 min)ª | | 16095 | 7 | 15196 | 6 | 15653 | 7 | 15534 | 7 | 13891 | 7 |
| APGAR <7 (5 min)ª | | 6359 | 3 | 5751 | 2 | 5709 | 2 | 5508 | 2 | 5356 | 3 |
| Neonatal death and stillbirthª (per 1000) | | 1920 | 8 | 1790 | 8 | 1725 | 7 | 1691 | 7 | 1420 | 7 |
| Maternal deathª (per 100000) | | 24 | 10 | 16 | 7 | 18 | 7 | 17 | 7 | 13 | 6 |

ªParticipants with missing values for age at delivery (n=1200), pre-pregnancy BMI (n=181037), fertility treatment (n=24028), number of fetuses (n=1449), mode of delivery (n=7505), HDP (n=3017), FGR (n=1587), gestational age at delivery (n=342), birth weight (n=783), APGAR <7 (1 min) (n=2077), PAGAR <7 (5 min) (n=3139), neonatal death and stillbirth (n=1532), and maternal death (n=487) are excluded.

BMI: body mass index, HDP: hypertensive disorder of pregnancy, FGR: fetal growth restriction

Supplementary Table S2. Impact of the pandemic on pregnancy complications and delivery outcomes by number of fetuses (During=2020.4-12 vs. Before=2016-2019)

|  |  | During (n=152301) vs. Before (n=955780) the pandemic | | | | | |
| --- | --- | --- | --- | --- | --- | --- | --- |
|  |  | Univariate analysis | | | Multivariate analysis | | |
|  |  | cOR^a^ | 95% CI | p value | aOR^a^ | 95% CI | p value |
| **Singleton pregnancy (n=1037480)** | | | | | | | |
| Pregnancy complications | | | | | | | |
| HDP (n=1034865) | | 1.102 | 1.077–1.127 | <0.001^b^ | 1.064^f^ | 1.035–1.094 | <0.001^b,d^ |
| FGR (n=1036244) | | 1.026 | 0.996–1.058 | 0.086^b^ | 1.045^g^ | 1.009–1.083 | 0.015^b,d^ |
| Delivery outcomes | | | | | | | |
| Gestational age at delivery  (n=1037175) | Preterm | 0.981 | 0.963–0.999 | 0.034^c^ | 0.994^h^ | 0.972-1.017 | 0.608^c,d^ |
|  | Post-term | 0.780 | 0.674–0.903 | <0.001^c^ | 0.731^h^ | 0.610-0.876 | <0.001^c,d^ |
| Birth weight  (n=1037064) | Low | 0.974 | 0.959–0.990 | 0.001^c^ | 0.983^h^ | 0.962-1.004 | 0.108^c,d^ |
|  | High | 1.030 | 0.969–1.095 | 0.337^c^ | 0.972^h^ | 0.901-1.048 | 0.461^c,d^ |
| APGAR <7 (1 min) (n=1035651) | | 1.060 | 1.036–1.085 | <0.001^b^ | 1.065^i^ | 1.031-1.097 | <0.001^b,d^ |
| APGAR <7 (5 min) (n=1034631) | | 1.089 | 1.050–1.129 | <0.001^b^ | 1.098^i^ | 1.046-1.152 | <0.001^b,d^ |
| Neonatal death and stillbirth (n=1037258) | | 0.957 | 0.893–1.026 | 0.217^b^ | 0.990^i^ | 0.902-1.087 | 0.835^b,d^ |
| Maternal death (n=1037253) | | 0.982 | 0.521–1.854 | 0.956^b^ | 1.410^i^ | 0.659-3.020 | 0.376^b,d^ |
| **Multiple pregnancy (n=70601)** | | | | | | | |
| Pregnancy complications | | | | | | | |
| HDP (n=70401) | | 0.918 | 0.857–0.984 | 0.015^b^ | 0.932^f^ | 0.856–1.016 | 0.108^b,e^ |
| FGR (n=70452) | | 0.964 | 0.896–1.038 | 0.329^b^ | 1.102^g^ | 1.009–1.204 | 0.031^b,e^ |
| Delivery outcomes | | | | | | | |
| Gestational age at delivery  (n=70587) | Preterm | 0.853 | 0.819–0.889 | <0.001^c^ | 1.026^h^ | 0.973-1.082 | 0.347^c,e^ |
|  | Post-term | 7.693 | 1.285–46.050 | 0.025^c^ | 7.397^h^ | 1.036-52.822 | 0.046^c,e^ |
| Birth weight  (n=70281) | Low | 0.736 | 0.705–0.770 | <0.001^c^ | 0.980^h^ | 0.923-1.040 | 0.507^c,e^ |
|  | High | 8.998 | 2.708–29.897 | <0.001^c^ | 1.753^h^ | 0.195-15.776 | 0.617^c,e^ |
| APGAR <7 (1 min) (n=70372) | | 0.925 | 0.869–0.985 | 0.015^b^ | 0.988^i^ | 0.909-1.074 | 0.776^b,e^ |
| APGAR <7 (5 min) (n=70327) | | 0.895 | 0.811–0.988 | 0.028^b^ | 0.947^i^ | 0.829-1.081 | 0.418^b,e^ |
| Neonatal death and stillbirth (n=69639) | | 0.847 | 0.710–1.010 | 0.064^b^ | 0.857^i^ | 0.661-1.111 | 0.244^b,e^ |
| Maternal death (n=70484) | | NA | NA | NA | NA | NA | NA |

^a^cOR and aOR mean crude and adjusted odds ratios analyzed with univariate and multivariate logistic regression models.

^b^Binary logistic regression.

^c^Multinominal logistic regression analysis.

^d^Participants with missing values for HDP (n=271416), FGR (n=271416), gestational age at delivery (n=271587), birth weight (n=271618), APGAR <7 (1 min) (n=278499), APGAR <7 (5 min) (n=279459), neonatal death and stillbirth (n=277539), and maternal death (n=277523) are excluded.

^e^Participants with missing values for HDP (n=19145), FGR (n=19145), gestational age at delivery (n=19153), birth weight (n=19363), APGAR <7 (1 min) (n=19701), APGAR <7 (5 min) (n=19738), neonatal death and stillbirth (n=19625), and maternal death (n=19615) are excluded.

^f^Adjusted for age at delivery, delivery location, pre-pregnancy BMI, fertility treatment, and FGR.

^g^Adjusted for age at delivery, delivery location, pre-pregnancy BMI, fertility treatment, and HDP.

^h^Adjusted for age at delivery, delivery location, pre-pregnancy BMI, fertility treatment, HDP, and FGR.

^i^Adjusted for age at delivery, delivery location, pre-pregnancy BMI, fertility treatment, mode of delivery, HDP, FGR, gestational age at delivery, and birth weight.

CI: confidence interval, HDP: hypertensive disorder of pregnancy, FGR: fetal growth restriction
